# Supplementary material for: Cytology and HPV Co-Testing for Detection of Vaginal Intraepithelial Neoplasia: A Retrospective Study
Source: Cancers (Basel). 2023 Sep 19;15(18):4633. doi: 10.3390/cancers15184633 (PMC10526267; doi:10.3390/cancers15184633)
Supplement: Supplementary file 1 [file cancers-15-04633-s001.zip › cancers-2500175-supplementary.pdf]

Supplement

Table S1. Pap smear versus histology (n = 279).

Pap smear	Bethesda	Benign (n = 86)	VaIN I/LSIL (n = 116)	VaIN II/HSIL (n = 41)	VaIN III/HSIL (n = 33)	Carcinoma (n = 3)
I (n = 65)	NILM	29 (44.6%)	33 (50.8%)	2 (3.1%)	1 (1.5%)	0
II-a (n = 42)	NILM	21 (50%)	18 (42.9%)	2 (4.8%)	1 (2.4%)	0
II-p (n = 17)	ASC-US	6 (35.3%)	8 (47.1%)	2 (11.8%)	1 (5.9%)	0
IIID1 (n = 70)	LSIL	17 (24.3%)	36 (51.4%)	11 (15.7%)	6 (8.6%)	0
IIID2 (n = 38)	HSIL	2 (5.3%)	11 (28.9%)	19 (50%)	6 (15.8%)	0
III-g (n = 1)	AGC, endocervical favoring neoplasia	1 (100%)	0	0	0	0
III-p (n = 24)	ASC-H	8 (33.3%)	10 (41.6%)	2 (8.3%)	3 (12.5%)	1 (4.2%)
IVa-p (n = 19)	HSIL	2 (10.5%)	0 (0%)	3 (15.7%)	14 (73.7%)	0
IVb-p (n = 1)	HSIL with features suspicious for invasion	0	0	0	1 (100%)	0
V-p (n = 2)	Squamous cell carcinoma	0	0	0	0	2 (100%)

AGC, atypical glandular cells; ASC-H, atypical squamous cells, cannot exclude HSIL; ASC-US, atypical squamous cells of undetermined significance; HSIL, high-grade squamous intraepithelial lesion; LSIL, low-grade squamous intraepithelial lesion; NILM, negative for intraepithelial lesion or malignancy; VaIN, vaginal intraepithelial neoplasia.

Table S2. Pap smear versus histology (after hysterectomy; n = 153).

Pap smear	Bethesda	Benign (n = 45)	VaIN I/LSIL (n = 62)	VaIN II/HSIL (n = 22)	VaIN III/HSIL (n = 22)	Carcinoma (n = 2)
I (n = 39)	NILM	18 (46.1%)	18 (46.1%)	2 (5.1%)	1 (2.6%)	0
II-a (n = 23)	NILM	10 (43.5%)	11 (47.8%)	1 (4.3%)	1 (4.3%)	0
II-p (n = 8)	ASC-US	3 (37.5%)	4 (50.0%)	0 (0%)	1 (12.5%)	0
IIID1 (n = 33)	LSIL	7 (21.2%)	19 (57.6%)	5 (15.2%)	2 (6.1%)	0
IIID2 (n = 24)	HSIL	0	7 (29.2%)	12 (50%)	5 (20.8%)	0
III-g (n = 0)	AGC, endocervical favoring neoplasia	0	0	0	0	0
III-p (n = 12)	ASC-H	6 (50.0%)	3 (25%)	1 (8.3%)	1 (8.3%)	1 (8.3%)
IVa-p (n = 13)	HSIL	1 (7.7%)	0	1 (7.7%)	11 (84.6%)	0
IVb-p (n = 0)	HSIL with features suspicious for invasion	0	0	0	0	0
V-p (n = 1)	Squamous cell carcinoma	0	0	0	0	1 (100%)

AGC, atypical glandular cells; ASC-H, atypical squamous cells, cannot exclude HSIL; ASC-US, atypical squamous cells of undetermined significance; HSIL, high-grade squamous intraepithelial lesion; LSIL, low-grade squamous intraepithelial lesion; NILM, negative for intraepithelial lesion or malignancy; VaIN, vaginal intraepithelial neoplasia.

Table S3. Pap smear versus histology (without hysterectomy; n = 126).

Pap smear	Bethesda	Benign (n = 41)	VaIN I/LSIL (n = 54)	VaIN II/HSIL (n = 19)	VaIN III/HSIL (n = 11)	Carcinoma (n = 1)
I (n = 26)	NILM	11 (42.3%)	15 (57.7%)	0	0	0
II-a (n = 19)	NILM	11 (57.9%)	7 (36.8%)	1 (5.3%)	0	0
II-p (n = 9)	ASC-US	3 (33.3%)	4 (44.4%)	2 (22.2%)	0	0
IIID1 (n = 37)	LSIL	10 (27.0%)	17 (45.9%)	6 (16.2%)	4 (10.8%)	0
IIID2 (n = 14)	HSIL	2 (11.1%)	4 (38.9%)	7 (38.9%)	1 (11.1%)	0
III-g (n = 1)	AGC, endocervical favoring neoplasia	1 (100%)	0	0	0	0
III-p (n = 12)	ASC-H	2 (16.7%)	7 (58.3%)	1 (8.3%)	2 (16.7%)	0

IVa-p (n = 6)	HSIL	1 (16.7%)	0 (0%)	2 (33.3%)	3 (50%)	0
IVb-p (n = 1)	HSIL with features suspicious for invasion	0	0 (0%)	0 (0%)	1 (100%)	0
V-p (n = 1)	Squamous cell carcinoma	0	0	0	0	1 (100%)

AGC, atypical glandular cells; ASC-H, atypical squamous cells, cannot exclude HSIL; ASC-US, atypical squamous cells of undetermined significance; HSIL, high-grade squamous intraepithelial lesion; LSIL, low-grade squamous intraepithelial lesion; NILM, negative for intraepithelial lesion or malignancy; VaIN, vaginal intraepithelial neoplasia.
